# Cognitive Behavioral Therapy vs. Eye Movement Desensitization and Reprocessing for Treating Panic Disorder: A Randomized Controlled Trial

**DOI:** 10.3389/fpsyg.2017.01409

**Published:** 2017-08-18

**Authors:** Ferdinand Horst, Brenda Den Oudsten, Wobbe Zijlstra, Ad de Jongh, Jill Lobbestael, Jolanda De Vries

**Affiliations:** ^1^Department of Psychiatry, St. Elisabeth Hospital Tilburg, Netherlands; ^2^Department of Medical and Clinical Psychology, Centre of Research on Psychology in Somatic Diseases Tilburg, Netherlands; ^3^Department of Education and Research, St. Elisabeth Hospital Tilburg, Netherlands; ^4^Department of Behavioral Science, Academic Centre for Dentistry Amsterdam, University of Amsterdam and VU University Amsterdam, Netherlands; ^5^School of Health Sciences, Salford University Manchester, United Kingdom; ^6^Institute of Health and Society, University of Worcester Worcester, United Kingdom; ^7^Department of Clinical Psychological Science, Maastricht University Maastricht, Netherlands; ^8^Department of Medical Psychology, St. Elisabeth Hospital Tilburg, Netherlands

**Keywords:** EMDR, CBT, Panic disorder, psychotherapy, RCT

## Abstract

**Objective:** Cognitive Behavioral Therapy (CBT) is an effective intervention for patients with panic disorder (PD). From a theoretical perspective, Eye Movement Desensitization and Reprocessing (EMDR) therapy could also be useful in the treatment of PD because: (1) panic attacks can be experienced as life threatening; (2) panic memories specific to PD resemble traumatic memories as seen in posttraumatic stress disorder (PTSD); and (3) PD often develops following a distressing life event. The primary objective of this Randomized Controlled Trial (RCT), was to compare EMDR therapy with CBT for PD and determine whether EMDR is not worse than CBT in reducing panic symptoms and improving Quality Of Life (QOL).

**Methods:** Two-arm (CBT and EMDR) parallel RCT in patients with PD (*N* = 84). Patients were measured at baseline (T1), directly after the last therapy session (T2), and 3 months after ending therapy (T3). Non-inferiority testing (linear mixed model with intention-to-treat analysis) was applied. Patients were randomly assigned to 13 weekly 60-min sessions of CBT (*N* = 42) or EMDR therapy (*N* = 42). Standard protocols were used. The primary outcome measure was severity of PD at T3, as measured with the Agoraphobic Cognitions Questionnaire (ACQ), the Body Sensations Questionnaire (BSQ), and the Mobility Inventory (MI). The secondary outcome measure was QOL, as measured with the World Health Organization Quality of Life short version (WHOQOL-Bref), at T3.

**Results:** The severity of PD variables ACQ and BSQ showed non-inferiority of EMDR to CBT, while MI was inconclusive (adjusted analyses). Overall QOL and general health, Psychological health, Social relationships, and Environment showed non-inferiority of EMDR to CBT, while Physical health was inconclusive.

**Conclusion:** EMDR therapy proved to be as effective as CBT for treating PD patients.

**Trial Registration:** Dutch Trial Register, Nr. 3134 http://www.trialregister.nl/trialreg/admin/rctview.asp?TC=3134

## Introduction

Panic disorder (PD) is characterized by recurrent, unexpected panic attacks and hyperarousal symptoms such as palpitations, pounding heart, chest pain, sweating, trembling, or shaking (Frances, 2004). These symptoms are often experienced as catastrophic and can have a great impact on daily life (Frances, 2004). Prevalence rates of PD are around 2.1% (Batelaan et al., 2006). Women are twice as likely to develop PD compared to men. Up to 50% of patients meet the criteria of agoraphobia (Weissman et al., 1997). In addition, widowed, lower educated, and divorced persons are more likely to experience panic attacks (Batelaan et al., 2006).

Several controlled treatment effect studies have shown that cognitive behavioral therapy (CBT), particularly interoceptive exposure, is the most effective intervention for PD (Barlow et al., 1989, 2000; Öst et al., 2004; Furukawa et al., 2007). Typical for this approach is that patients are exposed to exercises that evoke the physical sensations associated with a panic attack, such as hyperventilation, in order to experience that the worst expected outcome (e.g., dying) does not occur (i.e., “expectancy violation”). Approximately 40-90% of patients treated with CBT are panic free directly after treatment (Bakker et al., 1999). Variations in treatment effects are strongly determined by the selected study population (e.g., with/without comorbidity) and the content of CBT (e.g., whether in vivo exposure is offered) (Bakker et al., 1999; Rief et al., 2000). Furthermore, several studies have shown that the quality of life (QOL) for patients with PD improves after CBT (Telch et al., 1995; Davidoff et al., 2012). Nevertheless, a group of patients still needs additional treatment after CBT because some patients do not benefit, while others do not make a full recovery or develop other affective disorders (Van Balkom et al., 1996; Bakker et al., 1999). Eye Movement Desensitization and Reprocessing (EMDR) therapy is a treatment procedure for patients who suffer from past traumatic experiences in the present (Shapiro, 2002). In EMDR therapy the focus is on resolving disturbing memories of distressing or traumatic events by focusing on the memory while making eye movements at the same time. Besides CBT, EMDR is recommended as a first-line treatment for psychological trauma (Bisson et al., 2007). Despite the well-examined efficacy of EMDR for Post-Traumatic Stress Disorder (PTSD), the applicability of EMDR for other anxiety disorders, like PD, has hardly been examined (De Jongh and ten Broeke, 2009). There are several reasons why EMDR could be useful in the treatment of PD. Firstly, panic attacks likely occur unexpectedly, are experienced as distressing, cause a subjective response of fear or helplessness, and can be considered life threatening (McNally and Lukach, 1992; Hagenaars et al., 2009). Secondly, there are indications that panic memories in PD resemble traumatic memories as seen in PTSD (Hagenaars et al., 2009). Thirdly, there are indications that PD often develops after one or more distressing life events (Faravelli and Pallanti, 1989; Horesh et al., 1997). The few available studies on EMDR as PD treatment (Goldstein and Feske, 1994; Feske and Goldstein, 1997; Goldstein et al., 2000), all performed by the same research group, found a decrease in panic complaints and anticipatory anxiety in most EMDR-treated patients (Goldstein and Feske, 1994). Goldstein et al. (2000) showed that EMDR was superior to the waitlist condition on panic and agoraphobia severity, albeit no significant change was apparent on cognitive measures or on panic attack frequency. Importantly, these studies only included a short EMDR procedure and some essential parts of the current EMDR protocol (e.g., the installation of a “future template”) were lacking (De Jongh and ten Broeke, 2009). More recently, a pilot study comparing 12 sessions of EMDR to CBT for PD, found no differences between both treatments, except that EMDR resulted in significantly less frequent panic attacks (Faretta, 2013). Although the effect of EMDR on QOL in PD patients was not examined, QOL seems to be an important outcome measure as PD is a very stressful condition (Trompenaars et al., 2005).

In conclusion, CBT has been found to be effective for a considerable number of patients suffering from PD. The treatment of PD with EMDR seems plausible, but previous studies are limited and replications are needed. This is the first randomized controlled trial (RCT) that directly compares CBT and EMDR therapy in PD patients regarding PD severity and QOL.

The primary aim of this RCT was to examine if EMDR therapy is not worse than CBT among patients with PD on symptom severity and QOL 3 months post-treatment. It is hypothesized that EMDR is not worse than CBT.

## Materials and methods

### Design

The study was approved by the Medical Ethical Board of the St. Elisabeth hospital in Tilburg, the Netherlands and was registered in the Dutch Trial Register (www.trialregister.nl, NTR 3134). All included patients gave their written consent before enrollment. This study is a two-arm parallel RCT, including CBT and EMDR therapy.

### Participants

Patients were recruited, assessed, and treated at the department of psychiatry, St. Elisabeth hospital, Tilburg, the Netherlands between February 2010 and December 2013. Advertisements were placed in a local newspaper to inform people about the existence of our study. When someone wanted to participate he or she had to visit his or her general practitioner. Patients were referred to the hospital by general practitioners.

Inclusion criteria were: (1) age between 18 and 65 years old; (2) the presence of a SCID-I primary diagnosis of PD (First et al., 1997); and (3) sufficient knowledge of the Dutch language.

Exclusion criteria were: (1) comorbid diagnosis of dementia, psychosis, severe depression, bipolar disorder, and/or another psychiatric disorder that was more prominent than the PD; (2) use of more than 20 standard units of alcohol a week; and (3) use of benzodiazepines and/or other sedative agents (De Jongh and ten Broeke, 2006). This last criterion was added because benzodiazepines or other sedative agents are likely to interfere with the level of arousal that is needed for EMDR therapy to be effective (Little et al., 2017). Patients who use modern antidepressants (e.g., Selective Serotonin Reuptake Inhibitors (SSRIs) or Serotonin and Norepinephrine Reuptake Inhibitors (SNRIs) and/or classic antidepressants (e.g., Tricyclic Antidepressants (TCA) were required to be on a stable medication dose (i.e., unchanged dosage of medication), 6 weeks prior to trial until the end. Patients were not allowed to attend any form of therapy during the whole trial. Patients not eligible for participation were offered treatment as usual.

### Measures

The primary outcome measure was the severity of the PD, assessed with the Agoraphobic Cognitions Questionnaire (ACQ), which measures the degree of catastrophic cognitions when feeling anxious or tense (Chambless et al., 1984). The two subscales have a good internal consistency. The discriminant validity and construct validity are also good (Chambless et al., 1985).

The Body Sensations Questionnaire (BSQ) measures anxiety about bodily sensations and consists of two questionnaires; while the BSQ1 assesses the amount of fear, the BSQ2 measures how often the sensations are experienced when the patient feels anxious or tense (Chambless et al., 1984). The internal consistency and the test-retest reliability of the BSQ are good. Furthermore, the BSQ has good discriminant- and construct validity (Chambless et al., 1985).

The Mobility Inventory (MI) measures the degree to which places or situations are avoided with a trusted companion (MI-ac) and when the patient is alone (MI-al) (Chambless et al., 1985). Both subscales have a good internal consistency, discriminant validity and construct validity (Chambless et al., 1985). For ACQ, BCQ, and MI, lower scores indicate better outcomes.

The secondary outcome measure, QOL, was assessed with the World Health Organization Quality of Life short version (WHOQOL-Bref) (De Vries and van Heck, 1995). This measure consists of one generic facet (Overall quality of life and general health) and four domains (i.e., “Physical health,” “Psychological health,” “Social relationships,” and “Environment”) (De Vries and van Heck, 1995). Higher scores indicate better QOL. The WHOQOL-Bref is sensitive for changes over time and for treatment influences. The psychometric properties of the WHOQOL-Bref are also good (Trompenaars et al., 2005).

### Procedure

All patients were first screened by a psychiatrist who conducted a regular psychiatric interview, including the registration of the participants' medical status and medication use. Participation was voluntary and patients could withdraw from the study at any time without specifying a reason. After referral by a psychiatrist and before randomization, patients were screened with the Structured Clinical Interview for DSM-IV Axis I disorders (SCID-I) (First et al., 1997). The SCID-I was conducted by independent clinicians who were trained intensively during a 2-day workshop.

Patients eligible for participation were randomized to one of two treatment groups. Randomization was carried out by an independent secretary, who had 84 sealed envelopes, of which 42 contained a note with “EMDR” written on it, and 42 included a note with “CBT” on it. In both groups, a standardized treatment protocol was used. For each eligible patient, random assignment of sealed envelopes was performed. Before randomization, patients signed an informed consent. Patients were measured at baseline (T1), post-treatment (T2), and 3 months follow-up (T3), and received no financial compensation for participation.

### Treatment

In total, six licensed clinical psychologists (three men, three women) performed the EMDR and CBT treatments. In both groups, standardized treatment protocols were used. Therapists who performed EMDR therapy (one man, one woman) were both accredited practitioners by the European association. Therapists performing CBT treatment (three men, two women) were accredited CBT therapists by the Dutch National CBT Association.

The CBT protocol is the Dutch version of Craske and Barlow's (2008) and consists of 13 weekly sessions lasting about 60 min each (Craske and Barlow, 2008). During the first part (psycho-education), the patient is informed about panic attacks and PD. The second part consists of teaching and applying relaxation exercises which help the patient to reduce general anxiety. The third part consists of interoceptive exposure exercises in order to become accustomed to, and to cope with, the fear of bodily sensations. The fourth part is cognitive therapy in which the patients learn to recognize their automatic, anxious thoughts and formulate alternative, more adaptive thoughts. Finally, *in vivo* exposure consisted of learning patients to cope with the anxiety experienced during situations or activities that are feared and avoided by using an anxiety hierarchy (Kampman et al., 2004).

The EMDR treatment protocol is the Dutch version (De Jongh and ten Broeke, 2006) of Shapiro's EMDR protocol (Shapiro, 2001) and consists of 13 weekly sessions lasting about 60 min each. In this protocol, a patient is first informed about EMDR therapy, traumatic memories are identified, and the course of current symptoms is evaluated. In the present study the case conceptualization was conducted according to the “first method” of the “Two Method Approach” that deals with symptoms whereby memories of the etiological and/or aggravating events were meaningfully specified on a time line. To this end, the memories of the distressing events that were assumed to play a key role in the acquisition and maintenance of the condition and evoked distress, were determined. Subsequently, the memories that evoked the most disturbance, e.g., the first or worst panic attack, were reprocessed first using working memory taxation by listening to alternating audio tones. Subsequently, other memories that were considered to contribute to a patient's current symptoms were targeted in the same way (De Jongh et al., 2010). During EMDR therapy, patients are asked to report what associations come to mind and the patient is guided to refocus on that association. This is continued until the patient no longer reports any distress related to the target image. Afterwards, the patient is asked to formulate a positive belief regarding the target image.

### Supervision and treatment integrity

To each treatment group, 20 h of group supervision by an independent qualified EMDR or CBT supervisor were given. Additional supervision by telephone or e-mail was provided on request. All patients were asked permission to make video recordings of the treatment sessions, to ensure that therapists adhered to the treatment protocol. During the study, therapists had supervision sessions in which adherence to the therapist protocol was evaluated and discussed to maintain quality and homogeneity of the intervention protocol.

### Statistical analysis

According to the method of Faul et al. (2009), a sample size calculation was performed using G-Power 3.1.7 which showed that in total, 102 patients would be needed (non-inferiority test, effect size Cohen's *d* = 0.5, one-sided alpha = 0.05, power = 0.80). Anticipating 20% drop out, 128 patients were needed. For each outcome variable, linear mixed models (with ML estimation) were specified including main effects of group, time (categorical), and interaction effect group^*^time. The dependence of the repeated measures was taken into account by using the unstructured error covariance pattern model. Covariates (i.e., age, gender, education, marital status, duration of complaints, number of axis I diagnoses, received previous treatment, and antidepressant treatment) were added to obtain adjusted results under the missing at random assumption.

Non-inferiority testing was used to determine whether EMDR is not worse than CBT (Piaggio et al., 2006, 2012). For ACQ, BCQ, and MI, the null hypothesis is (EMDR - CBT) > δ, and the alternative hypothesis is (EMDR - CBT) ≤ δ, where δ is the margin that is set at minimal clinical relevance. If the upper bound of confidence interval of 90% is below δ, it is concluded that EMDR is non-inferior to CBT. For ACQ and BCQ, the margin was δ = 5, and for MI, the margin was δ = 8. The margins of these questionnaires were determined by clinical experts. For WHOQOL-BREF, the non-inferiority was reversed and the margin was δ = −1 (Den Oudsten et al., 2013).

Group differences were analyzed at T3. Intention-to-treat approach was used on the patients that started treatment, while per-protocol approach was used as a sensitivity analysis on patients that completed all treatments (Piaggio et al., 2006, 2012). For effect size measure, Cohen's d was computed as mean difference divided by baseline pooled standard deviation. Statistical analyses were performed in SPSS version 19.0.

## Results

**Figure 1:**
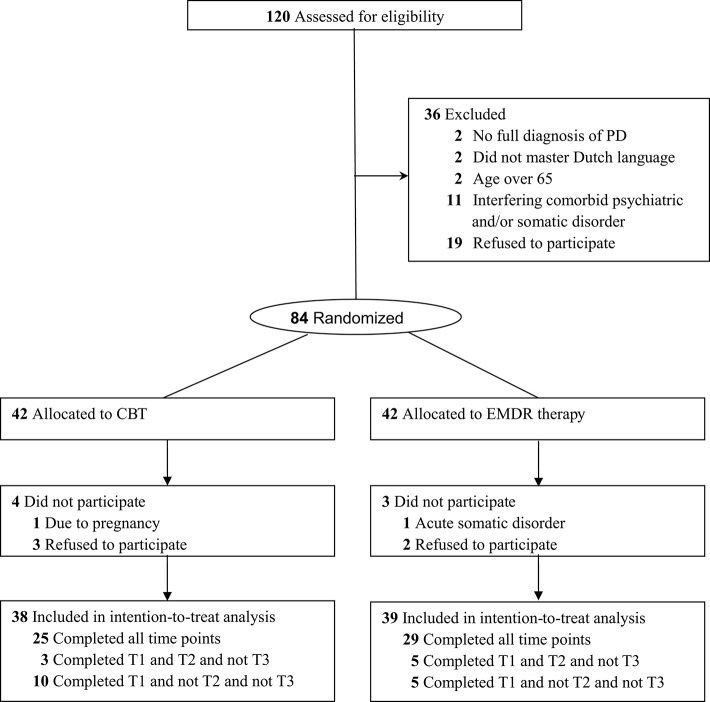
Flow of participants through the trial.

Figure 1 shows the patient flow through the trial. Despite an extended inclusion period, in total, 120 patients could be assessed for eligibility, from which 36 were excluded. Accordingly, were randomized to both treatment groups: 42 patients to CBT and 42 to EMDR therapy. Table 1 displays the baseline and clinical characteristics of both groups. No significant differences in age, gender, education, marital status, and number of axis I diagnoses at baseline were found. However, patients in the CBT group had experienced significantly shorter duration of PD and received significantly less previous treatment than those in the EMDR group. Significantly more patients in the EMDR group received antidepressant treatment than those in the CBT group.

**Table 1:** Baseline demographic and clinical characteristics.

**Characteristics**	**CBT (*N* = 42)**	**EMDR (*N* = 42)**	**Total sample (*N* = 84)**	***P***
Age, mean (SD), year	40.9 (12.1)	37.0 (10.7)	39.0 (11.5)	0.126^a^
Gender, No.				0.491^b^
Male	16	13	29	
Female	26	29	55	
Education, No. (%)				0.143^b^
Low (<10 years)	9 (21%)	13 (31%)	22 (26%)	
Middle (10–14 years)	24 (57%)	15 (36%)	39 (46%)	
High (>14 years)	9 (21%)	14 (33%)	23 (27%)	
Marital status, No. (%)				0.078^b^
Unmarried	20 (48%)	28 (67%)	48 (57%)	
Married	22 (52%)	14 (33%)	36 (43%)	
Duration of PD, No. (%)				0.027^b^^*^
<2 years	25 (60%)	12 (29%)	37 (44%)	
2–5 years	8 (19%)	12 (29%)	20 (24%)	
5–10 years	2 (5%)	8 (20%)	10 (12%)	
>10 years	7 (17%)	9 (22%)	16 (19%)	
Missing	0	1	1 (1%)	
DSM-IV-TR Axis I diagnoses^a^, mean (SD)	2.5 (1.1)	2.5 (1.1)	2.5 (1.1)	0.766^a^
Agoraphobia^I^, No. (%)	33 (80%)	28 (68%)	61 (74%)	0.161^b^
Received previous treatment for PD, No. (%)				0.001^b^^**^
Yes	18 (43%)	33 (79%)	51 (61%)	
No	24 (57%)	9 (21%)	33 (39%)	
Received antidepressant treatment No. (%)				0.026^b^^*^
Yes	12 (29%)	22 (52%)	34 (40%)	
No	30 (71%)	20 (48%)	50 (60%)	

*CBT, cognitive behavioral therapy; EMDR, eye movement desensitization and reprocessing; PD, Panic Disorder; SCID-I, Structured Clinical Interview for DSM-IV Axis I disorders; SD, standard deviation*.

I *Measured using SCID-I*.

a *Independent two-sampled t-test*.

b *Pearson Chi-Square*.

* *p < 0.05*.

** *p < 0.01*.

Seven patients (8%) did not start the first treatment session and were unaware of treatment allocation (Figure 1). Completers of all time points did not significantly differ from non-completers (i.e., missing at least one time point) on gender, education, and years of complaints. No unintended effects were found in both treatment groups.

### Primary outcome measures

**Figure 2:**
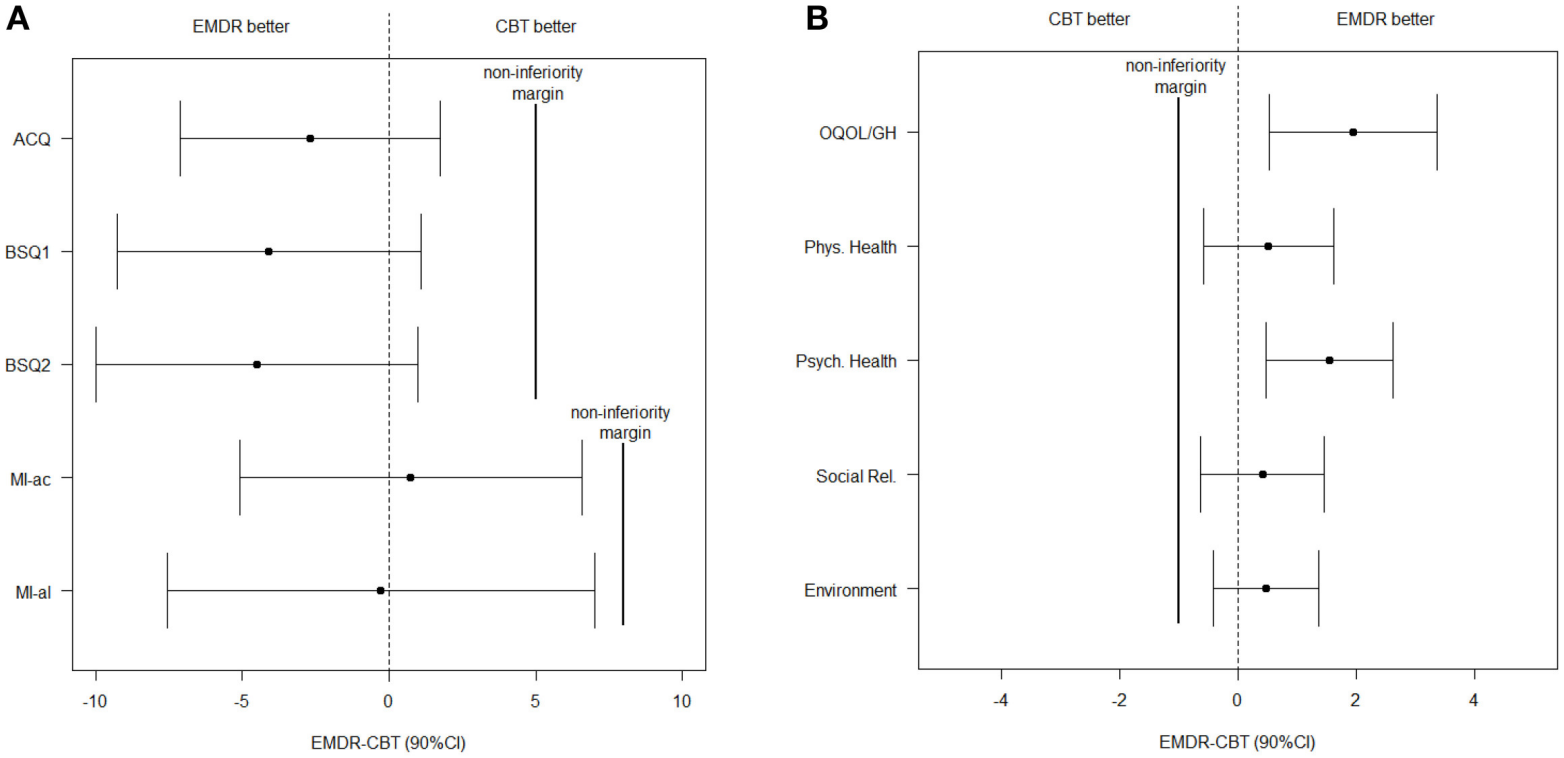
Unadjusted effects and 90% CI of **(A)** the symptoms and **(B)** the quality of life facet and domains at T3. ACQ, Agoraphobic Cognitive Questionnaire; BSQ1, Body Symptoms Questionnaire (amount of fear); BSQ2, Body Symptoms Questionnaire (how often sensations are experienced); CBT, Cognitive Behavioral Therapy; CI, Confidence Interval; EMDR, Eye Movement Desensitization and Reprocessing; MI-ac, Mobility Inventory (when accompanied); MI-al, Mobility Inventory (when alone); QOL, Quality Of Life; OQOL/GH, Overall Quality Of Life and General Health.

Information on observed outcome means and effect sizes for both treatment groups for all time points, are presented in Table 2. The intention-to-treat analyses at T3 were performed on 39 EMDR patients and 38 CBT patients. Scores on questionnaires measuring severity of PD (ACQ, BSQ1, BSQ2, MI-ac, and MI-al) showed non-inferiority of EMDR to CBT in the unadjusted analysis (Table 3, Figure 2A). In the adjusted analysis, this was also the case for ACQ, BSQ1, and BSQ2, whereas MI-ac and MI-al were inconclusive.

**Table 2:** Observed outcome means (standard deviation) for both treatment groups EMDR and CBT for baseline (T1), after treatment (T2), and 3 months follow up (T3).

	**CBT**					**EMDR**				
**Outcome**	**T1 (*N* = 38)**	**T2 (*N* = 28)**		**T3 (*N* = 25)**		**T1 (*N* = 39)**	**T2 (*N* = 34)**		**T3 (*N* = 29)**	
	**Mean (SD)**	**Mean (SD)**	***d*^a^**	**Mean (SD)**	***d*^b^**	**Mean (SD)**	**Mean (SD)**	***d*^a^**	**Mean (SD)**	**d^b^**
**SYMPTOMS^a^**										
ACQ	34.1 (9.6)	24.7 (8.8)	−0.86	27.5 (10.7)	−0.60	36.8 (12.1)	23.6 (10.5)	−1.21	25.1 (10.2)	−1.07
BSQ1	47.0 (11.8)	29.1 (9.4)	−1.44	34.1 (12.1)	−1.04	50.2 (13.0)	28.5 (10.4)	−1.74	30.2 (11.5)	−1.60
BSQ2	48.3 (11.2)	34.5 (9.9)	−1.25	40.3 (10.9)	−0.72	52.5 (10.7)	33.0 (12.4)	−1.77	36.3 (14.0)	−1.47
MI-ac	51.9 (18.8)	33.3 (9.7)	−0.99	35.2 (11.2)	−0.89	51.8 (19.1)	36.6 (16.9)	−0.80	36.2 (15.8)	−0.83
MI-al	62.2 (22.8)	41.3 (14.8)	−0.85	43.3 (17.3)	−0.77	68.1 (26.0)	42.0 (21.7)	−1.06	41.4 (17.5)	−1.09
**QOL^b^**										
OQOL/GH	10.8 (3.6)	14.4 (2.4)	1.00	13.0 (3.8)	0.62	10.6 (3.5)	14.7 (3.8)	1.16	15.3 (2.7)	1.33
Physical health	11.9 (2.6)	14.4 (2.4)	0.91	14.0 (2.7)	0.75	11.2 (3)	14.7 (3.1)	1.26	14.5 (2.5)	1.18
Psychological health	11.0 (2.5)	13.3 (1.8)	0.91	12.9 (2.7)	0.78	11.0 (2.6)	14.3 (2.8)	1.32	14.5 (2.1)	1.39
Social relationships	13.4 (2.8)	15.0 (2.6)	0.55	14.6 (2.3)	0.42	14.1 (3.0)	15.6 (3.0)	0.55	15.0 (2.5)	0.32
Environment	14.0 (2.3)	15.7 (1.9)	0.70	15.5 (2.1)	0.62	13.8 (2.5)	16.4 (2.4)	1.05	15.9 (1.9)	0.88

*ACQ, Agoraphobic Cognitive Questionnaire; BSQ1, Body Symptoms Questionnaire (amount of fear); BSQ2, Body Symptoms Questionnaire (how often sensations are experienced); CBT, Cognitive Behavioral Therapy; EMDR, Eye Movement Desensitization and Reprocessing; MI-ac, Mobility Inventory (when accompanied); MI-al, Mobility Inventory (when alone); QOL, Quality Of Life; OQOL/GH. Overall Quality Of Life and General Health; d, Mean difference divided by pooled (CBT + EMDR) baseline standard deviation; d^a^, d(T2-T1); d^b^, d(T3-T1)*.

**Table 3:** Non-inferiority effects EMDR vs. CBT at T3.

	**Unadjusted**			**Adjusted^f^**		
**Outcome**	**B (EMDR-CBT)**	**Lower 90%CI**	**Upper 90%CI**	**B (EMDR-CBT)**	**Lower 90%CI**	**Upper 90%CI**
**SYMPTOMS^a^**						
ACQ^c^	−2.68	−7.11	1.75^*^	−3.05	−7.92	1.82^*^
BSQ1^c^	−4.09	−9.26	1.08^*^	−3.40	−9.08	2.28^*^
BSQ2^c^	−4.50	−9.98	0.98^*^	−6.02	−11.97	−0.06^*^
MI-ac^d^	0.74	−5.09	6.58^*^	2.83	−3.61	**9.28**
MI-al^d^	−0.28	−7.56	7.00^*^	2.44	−5.21	**10.09**
**QOL^b^**						
OQOL/GH^e^	1.95	0.53^*^	3.37	1.25	−0.23^*^	2.74
Physical health^e^	0.51	−0.58^*^	1.61	−0.07	**−1.27**	1.13
Psychological health^e^	1.55	0.47^*^	2.62	1.41	0.29^*^	2.54
Social relationships^e^	0.41	−0.64^*^	1.45	0.47	−0.66^*^	1.60
Environment^e^	0.47	−0.42^*^	1.36	0.02	−0.97^*^	1.01

*ACQ, Agoraphobic Cognitive Questionnaire; B, unstandardized effect estimate; BSQ1, Body Symptoms Questionnaire (amount of fear); BSQ2, Body Symptoms Questionnaire (how often sensations are experienced); CBT, Cognitive Behavioral Therapy; CI, Confidence Interval; EMDR, Eye Movement Desensitization and Reprocessing; MI-ac, Mobility Inventory (when accompanied); MI-al, Mobility Inventory (when alone); QOL, Quality Of Life; OQOL/GH, Overall Quality Of Life and General Health*.

a *Lower scores indicates better for patient*.

b Higher scores indicates better for patient

c *Noninferiority test: **upper bound 90% CI** < 5*.

d *Noninferiority test: **upper bound 90% CI** < 8*.

e *Noninferiority test: **lower bound 90% CI** > (−1)*.

f *Adjusted for age, gender, education, marital status, duration of complaint, received previous treatment, number of axis I diagnoses and antidepressant*.

* *Indicates non-inferiority*.

### Secondary outcome measures

For the facet ‘Overall QOL and general health’ and the four QOL domains, EMDR appeared to be non-inferior to CBT at T3 in the unadjusted analysis (Table 3, Figure 2B). For the adjusted analyses, only “physical health” was inconclusive.

### Sensitivity analyses

Per-protocol analyses included the 62 patients that had T1 and T2 measurement (10 patients were removed in the CBT group and five in the EMDR group). All conclusions were similar to the intention-to-treat analyses, except for QOL domain “Environment” in which the unadjusted analysis at T3 was now inconclusive (lower bound −1.09).

## Discussion

This is the first RCT that tested whether EMDR is no worse than CBT (i.e., the “gold standard” for the treatment of PD). The results show that EMDR is no worse (i.e., non-inferior) than CBT with regard to severity of a wide range of PD symptoms, including anxiety related cognitions, fear of bodily sensations, as well as quality of life. Concerning the behavioral aspects of the condition, the tendency to avoid certain situations, the results were inconclusive. Intriguingly, despite both treatments were comparable in terms of effects, from face value the procedures seem to be opposed. That is, the CBT procedure for panic disorder entails specific exposures to patient's physical sensations (i.e., sensory experiences associated with anxiety, i.e., the conditioned stimuli), while disturbing memories of past events (the unconditioned stimuli, e.g., the first panic attack), that may have laid the groundwork for the panic disorder, are left untreated. In contrast, in EMDR therapy only memories of the latter type of events are targeted and processed, whereas the protocol only indirectly deals with the stimuli that normally would evoke a panic attack.

A strength of the current study is the use of manualized treatment protocols, including a relatively long therapeutic track consisting of 13 sessions making generalizability to clinical practice more feasible.

A limitation of the current study is the use of audio tones as the modality by which the memory taxation was performed. Laboratory studies provide evidence that audio tones are less optimal or appeared even less effective when compared to eye movements in diminishing the emotionality of memories underlying PTSD and other mental health problems (Van den Hout et al., 2012; De Jongh et al., 2013). This implies that when eye movements would have been applied in the present study the results might have been more profound. Furthermore, the determined sample size was not reached. Therefore, the study was underpowered given the expected effect size. Nonetheless, results showed larger effects sizes than a-priori expected, particularly for EMDR therapy. Concerning our randomization, it appeared that the two treatment groups differed on three aspects. Patients receiving CBT had a shorter PD duration, less previous treatment, and less antidepressant treatment compared to patients receiving EMDR. With regard the dropout rate, this was higher than expected, especially in the CBT group. This might partly be explained by the fact that Dutch law states that patients' decision to participate in scientific studies is voluntary, which means that patients may withdraw from the study at any time without specifying a reason. Therefore, we cannot provide a definite explanation for all patients. Another reason could be that patients who used benzodiazepines or other sedative agents were asked to stop medication so they could enter the study when clean. When patients asked for support, they were offered a clinical detox. Several patients refused to stop medication and therefore, received treatment as usual, and stopped participating in the study. Finally, no fidelity measure was used for CBT interoceptive exposure. To our knowledge, no such measure exists and developing and validating such a measure was beyond the scope of the current research. For EMDR fidelity measures do exist, but reporting this on its own seemed inappropriate.

Future long-term studies may provide more insight into the stability of the effects. This study has focused directly on comparing CBT with EMDR in the treatment of PD. Concerning the small sample size and the inconclusive results with regard to the MI, future studies may focus on combining both therapies, and especially on *in vivo* exposure with EMDR.

In conclusion, the present results provided evidence suggesting that EMDR therapy is as effective as CBT for patients with PD and may, therefore, be considered as a useful alternative to a conventional CBT treatment of PD patients.

## Author contributions

FH had full access to all the data in the study and takes responsibility for the integrity of the data and the accuracy of the data analyses. Study concept and design: FH, BD, WZ, AdJ, JL, and JD. Acquisition, analyses or interpretation of data: FH, BD, WZ, AdJ, JL, and JD. Drafting of the manuscript: FH, BD, WZ, AdJ, JL, and Jd. Critical revision of the manuscript for important intellectual content: FH, BD, WZ, AdJ, JL, and JD. Statistical analyses: FH, WZ, and JD. Administrative, technical, or material support: FH, BD, WZ, AdJ, JL, and JD.

## Study supervision

Veronique Boelaars (CBT) and Indra Spierts (EMDR).

### Conflict of interest statement

AdJ reported receiving income for published books or book chapters on EMDR and for training professionals in this method. The other authors declare that the research was conducted in the absence of any commercial or financial relationships that could be construed as a potential conflict of interest.
